# Decreased CXCR1 and CXCR2 expression on neutrophils in anti-neutrophil cytoplasmic autoantibody-associated vasculitides potentially increases neutrophil adhesion and impairs migration

**DOI:** 10.1186/ar3534

**Published:** 2011-12-08

**Authors:** Nan Hu, Johanna Westra, Abraham Rutgers, Berber Doornbos-Van der Meer, Minke G Huitema, Coen A Stegeman, Wayel H Abdulahad, Simon C Satchell, Peter W Mathieson, Peter Heeringa, Cees G M Kallenberg

**Affiliations:** 1 Department of Rheumatology and Clinical Immunology, University Medical Center Groningen, PO Box 30.001, 9700 RB, Groningen, The Netherlands; 2 Department of Nephrology, University Medical Center Groningen, PO Box 30.001, 9700 RB, Groningen, The Netherlands; 3 Department of Pathology and Medical Biology, University Medical Center Groningen, PO Box 30.001, 9700 RB, Groningen, The Netherlands; 4 Academic Renal Unit, University of Bristol, Second Floor, Learning and Research, Southmead Hospital, Bristol, BS10 5NB, UK

## Abstract

**Introduction:**

In anti-neutrophil cytoplasmic autoantibody (ANCA)-associated vasculitides (AAV), persistent inflammation within the vessel wall suggests perturbed neutrophil trafficking leading to accumulation of activated neutrophils in the microvascular compartment. CXCR1 and CXCR2, being major chemokine receptors on neutrophils, are largely responsible for neutrophil recruitment. We speculate that down-regulated expression of CXCR1/2 retains neutrophils within the vessel wall and, consequently, leads to vessel damage.

**Methods:**

Membrane expression of CXCR1/2 on neutrophils was assessed by flow cytometry. Serum levels of interleukin-8 (IL-8), tumor necrosis factor alpha (TNF-α), angiopoietin 1 and angiopoietin 2 from quiescent and active AAV patients and healthy controls (HC) were quantified by ELISA. Adhesion and transendothelial migration of isolated neutrophils were analyzed using adhesion assays and Transwell systems, respectively.

**Results:**

Expression of CXCR1 and CXCR2 on neutrophils was significantly decreased in AAV patients compared to HC. Levels of IL-8, which, as TNFα, dose-dependently down-regulated CXCR1 and CXCR2 expression on neutrophils *in vitro*, were significantly increased in the serum of patients with active AAV and correlated negatively with CXCR1/CXCR2 expression on neutrophils, even in quiescent patients. Blocking CXCR1 and CXCR2 with repertaxin increased neutrophil adhesion and inhibited migration through a glomerular endothelial cell layer.

**Conclusions:**

Expression of CXCR1 and CXCR2 is decreased in AAV, potentially induced by circulating proinflammatory cytokines such as IL-8. Down-regulation of these chemokine receptors could increase neutrophil adhesion and impair its migration through the glomerular endothelium, contributing to neutrophil accumulation and, in concert with ANCA, persistent inflammation within the vessel wall.

## Introduction

AAV comprises granulomatosis with polyangiitis (GPA), microscopic polyangiitis (MPA) and Churg Strauss syndrome (CSS), which share a spectrum of clinical manifestations reflecting necrotizing damage to small- and medium-sized vessels [1,2]. A role for neutrophils as effector cells in AAV is supported by a large body of evidence from *in vitro *and *in vivo *studies. After being primed by proinflammatory cytokines such as TNF-α, neutrophils can be activated by ANCA and release oxygen radicals and proteolytic enzymes, which have been shown to lyse endothelial cells in *in vitro *co-cultures [3,4]. *In vivo*, neutrophil accumulation in glomeruli has been observed in the early phase of crescentic glomerulonephritis and neutrophil depletion could completely prevent disease development in experimental models [5].

Migration of neutrophils is largely regulated by the concentration gradient of CXC-chemokines which contain a glutamic acid-leucine-arginine (ELR^+^) motif and are the most powerful chemoattractants for neutrophils. Interleukin 8 (IL-8) is the most potent member of the CXC family with high affinity for both of its receptors, CXCR1 and CXCR2, which are co-expressed on the membrane of neutrophils. Thus, binding of IL-8 to CXCR1/2 is a major element in neutrophil recruitment [6].

Neutrophils function in immune surveillance. Their activation, in terms of degranulation and oxidative burst, arms neutrophils with microbicidal activity, which normally appears only after they have migrated from the circulation and reach inflamed tissues [7]. However, in the pathology of AAV, necrotizing damage within the vessel wall suggests that ANCA-mediated neutrophil activation is already triggered during neutrophil recruitment. This may be caused by impaired neutrophil trafficking retaining activated neutrophils within the microvascular compartment.

Although consensus is still lacking whether ANCA bind to neutrophils in suspension, it has been well demonstrated that membrane bound-proteinase 3 (PR3) on neutrophils is significantly upregulated during neutrophil adhesion, and full neutrophil activation not only requires ANCA-antigen cross-linking but also adherence of neutrophils [8-11]. Here, we hypothesize that IL-8-CXCR1/CXCR2-mediated neutrophil recruitment is hampered in AAV, which leads to firm adhesion of neutrophils to the endothelium without further transmigration. Retained neutrophils within the microvascular compartment may be activated by ANCA, which will result in persistent inflammation of the vessel wall. To test this hypothesis, we investigated expression profiles of CXCR1 and CXCR2 on neutrophils in patients with AAV, related expression to levels of chemoattractants, and analyzed effects on transmigration.

## Materials and methods

### Patients and healthy controls

For measurement of CXCR1 and CXCR2 expression, 37 patients with quiescent AAV and 5 AAV patients with active disease were recruited from our out-patient clinic. Thirty healthy donors recruited from laboratory personnel were included as a normal control population. Characteristics of patients and controls are summarized in Table 1.

**Table 1:** Characteristics of patients and controls.

Characteristics	Number (%) or mean (range)			
	
	HC (*n *= 30)	AAV		
		
		Remission (*n *= 37)	Active^a^ (*n *= 5)	Active^b^ (*n *= 18)
Male	17 (57)	25 (68)	3 (60)	14 (78)
Age (years)	45 (28-64)	57 (26-84)	58 (47-68)	58 (32-81)
Diagnosis				
GPA		26	4	13
MPA		9	1	5
NCGN		2	0	0
ANCA-specificity				
PR3		24 (65)	4 (80)	13 (72)
MPO		13 (35)	1 (20)	5 (28)
Organ involvement				
ENT		0	4 (80)	5 (28)
Eye		0	1 (20)	4 (22)
Lung		0	4 (80)	8 (44)
Kidney		0	2 (40)	13 (72)
Skin		0	1 (20)	0
Nervous system		0	1 (20)	2 (11)
BVAS, median (range)		0	14.8 (6-26)	17.5 (6-33)
Treatment				
MMF		0	1 (20)	3 (17)
CYC		0	4 (80)	11 (61)
Prednisolon		0	4 (80)	16 (89)

**AAV**, ANCA-associated vasculitides; ANCA, anti-neutrophil cytoplasmic autoantibody; **BVAS**, Birmingham Vasculitis Activity Score; **CYC**, cyclophosphamide; **ENT**, ear, nose and throat; **GPA**, granulomatosis with polyangiitis; **HC**, healthy control; **MMF**, mycophenolate mophetil; **MPA**, microscopic polyangiitis; **MPO**, myeloperoxidase; **NCGN**, necrotizing crescentic glomerulonephritis; **PR3**, proteinase 3; ^a^Active patients included in CXCR1 and CXCR2 measurement by flow cytometry. ^b^Active patients included in cytokine profiling by ELISA.

A diagnosis of granulomatosis with polyangiitis (GPA), Churg Strauss syndrome (CSS) or microscopic polyangiitis (MPA) was based on the Chapel Hill definitions [12]. ANCA specificity for PR3 or myeloperoxidase (MPO) was determined by capture ELISA. Patients in remission were without treatment. Treatment of active patients is listed in Table 1. Disease severity was quantified using the Birmingham Vasculitis Activity Score (BVAS).

Sera from AAV patients and healthy volunteers were collected for cytokine profiling. For patients in remission, samples were drawn on the same day for CXCR1/2 measurement and cytokine testing. For patients who were in remission but had suffered from active disease before (*n *= 18), serum samples from active disease were retrieved from a stored serum bank. All sera were stored at -80°C before testing.

All subjects gave their informed consent and the study was approved by the hospital medical ethical committee.

### Antibodies used for flow cytometry

Peridin chlorophyll protein (PerCP)/Cy5.5-conjugated anti-CD14 (HCD14; Biolegend, San Diego, CA, USA) was used to discriminate neutrophils from monocytes in whole blood staining. Phycoerythrin (PE)-conjugated anti-CD181 (CXCR1, 5A12; BD Bioscience, Alphen aan de Rijn, The Netherlands), allophycocyanin (APC)-conjugated anti-CD182 (CXCR2, 6C6; BD Bioscience, Alphen aan de Rijn, The Netherlands) and fluorescein isothiocyanate (FITC)-conjugated anti-CD177 (NB1, MEM166; abCAM, Cambridge, UK) were used to detect membrane expression of CXCR1, CXCR2 and CD177, respectively. Irrelevant antibodies of the same isotype were used as negative controls.

### Cell culture

Human conditionally immortalized glomerular endothelial cells (CiGEnC) [13] were cultured in endothelial growth medium 2-microvascular (EGM2-MV; Cambrex-Lonza, Breda, The Netherlands) containing 5% FCS and growth factors, without vascular endothelial growth factor (VEGF), as supplement. CiGEnC up to passage 40 were propagated at 33°C to keep cells in a proliferative state. Experiments were carried out on cells that had grown into a confluent monolayer and had been incubated for 5 days at 37°C in order to acquire a non-proliferative/quiescent phenotype.

### Neutrophil isolation and stimulation

Neutrophils were isolated from peripheral blood according to routine procedures as described previously [14]. Briefly, heparinized venous blood was centrifuged on Lymphoprep (Axis-Shield, Oslo, Norway). Contaminating erythrocytes were lysed with ice-cold ammonium chloride buffer. Afterwards, cells were washed with cold Hanks' balanced salt solution (HBSS) without Ca^2+^/Mg^2+ ^(HBSS^-/-^) and resuspended in HBSS with Ca^2+^/Mg^2+ ^(HBSS^+/+^; GIBCO/Life Technologies, Breda, The Netherlands) to obtain 10^7 ^cells/ml.

Where indicated, cells were incubated with serial doses of interleukin-8 (IL-8; R&D Systems, Minneapolis, MN USA), angiopoietin-1 (ANGPT-1; R&D Systems, Minneapolis, MN USA), angiopoietin-2 (ANGPT-2; R&D Systems, Minneapolis, MN USA), recombinant human tumor necrosis factor-α (rhTNF-α; R&D Systems, Minneapolis, MN USA) or repertaxin (Sigma-Aldrich, ST. Louis, MO USA) at 37°C for 30 minutes. Non-stimulated cells were incubated with control medium under the same condition.

### Membrane staining of neutrophils for flow cytometry

Membrane expression of CXCR1, CXCR2 and CD177 on neutrophils was assessed by flow cytometry. According to the manufacturer's instructions, 100 μl of whole blood from patients and controls or 10^6 ^of purified neutrophils after *in vitro *stimulation were incubated with antibodies at room temperature, in the dark, for 15 minutes, followed by fixation and lysing of erythrocytes with 20 × volume of Fluorescence Activated Cell Sorting (FACS) lysing solution (BD Bioscience, San Jose, CA, USA). Fluorescence intensity was measured by FACS Calibur (Becton Dickinson Immunocytometry Systems, Mountain View, CA, USA) and calibrated using CellQuest™ software (Becton Dickinson). Results were analyzed using the Win-List software package (Verity Software House, Topsham, ME, USA). Mean fluorescence intensity (MFI) of 5.10^4 ^counted cells was measured in each sample. Neutrophils were gated as forward scatter^high ^(FSC^high^)-side scatter^high ^(SSC^high^)-CD14^low ^cells. Expression levels were presented as MFI corrected for nonspecific binding of isotype control antibodies on neutrophils from the same donor.

### MPO activity assay

Numbers of adherent or migrated neutrophils in adhesion or migration assay were quantified by myeloperoxidase (MPO) activity assay [15]. Briefly, cells were lysed with 0.5% Triton-X-100 at 4°C for 20 minutes and acidified by adding 1 M citrate buffer (pH = 4.2, 50 μl/1 ml of cell lysate) just prior to the reaction. Aliquots (75 μl) from each sample were transferred into wells of a flat-bottomed 96-well plate. 2.2'-azino-*bis*-3-ethylbenzothiazoline-6-sulfonic acid (ABTS) at a concentration of 0.05% in 100 m*M *citrate with 0.03% H_2_O_2 _was loaded in an equal volume as substrate for MPO. After the reaction, OD_405 _was measured using an ELISA plate reader. Numbers of neutrophils in each well were determined as based on standards generated from known concentrations of neutrophils from the same isolation.

### Neutrophil adhesion

Neutrophil adhesion to endothelial monolayers was quantified by adhesion assay as described previously with modifications [16]. CiGEnCs were seeded in 24-well plates and allowed to proliferate to confluence. Cell monolayers were carefully washed with HBSS^+/+ ^and stimulated with 10 ng/ml TNF-α at 37°C for 4 hours. Cells were then washed three times before 0.5 × 10^6 ^neutrophils were loaded. Where indicated, neutrophils were pretreated with serial doses of IL-8, TNF-α or repertaxin which is a noncompetitive allosteric inhibitor of CXCR1 and CXCR2 [17]. After incubation of endothelial cells with neutrophils at 37°C for 30 minutes, loosely adherent or non-adherent neutrophils were washed off with HBSS^-/- ^and the endothelial monolayers plus adherent neutrophils were lysed in 0.5% Triton-X-100. All experiments were run in duplicate. The numbers of neutrophils were quantified, as described above, by MPO activity assay.

### Neutrophil migration

Transwell inserts (Corning, NY, USA), with 3.0 μm pores in 12-well plates, were precoated with CiGEnCs. Confluence of the endothelial monolayers was determined by continued monitoring of trans-endothelial electrical resistance (TEER) [13] which plateaued at 20 to 25 Ω on the 5^th ^day after seeding. Histological staining of the membrane showed that the formation of monolayers on inserts required 6 × 10^4 ^cells and 5 days of culture. Therefore, all the Transwell inserts were coated with CiGEnCs following this standard procedure. Isolated neutrophils from healthy donors were preincubated with repertaxin, IL-8 or TNF-α, and 1.10^6 ^cells were loaded in the upper chamber and 10 ng/ml of IL-8, which showed optimal efficiency as a chemoattactant in titration in preliminary experiments (data not shown), was placed in the lower chamber. Wells without IL-8 in the lower chamber or neutrophils without any treatment in the upper chamber were included as controls. All experiments were run in duplicate. After co-incubation of neutrophils with the inserts at 37°C for 2 hours, the plates with inserts within the wells were centrifuged for 5 minutes at 50 g and 4°C to dislodge adherent neutrophils on the lower surface of the inserts. The cells in the lower chamber were collected and quantified by MPO activity assay.

### Serum levels of IL-8, TNF-α, angiopoietin-1 (ANGPT-1) and angiopoietin-2 (ANGPT-2)

Sera collected from AAV patients in remission (*n *= 37), patients with active disease (*n *= 18) and healthy blood donors (*n *= 30) were used for measurement of IL-8, TNF-α, ANGPT-1 and ANGPT-2. IL-8 and TNF-α levels were measured by ELISA (R&D Systems, Minneapolis, MN, USA) as described previously [18]. After sample incubation and binding of the biotinylated detecting antibodies, color reaction was performed with streptavidin-poly-HRP (Sanquin, Amsterdam, The Netherlands) and tetramethyl-benzidin (TMB, Roth, Karlsruhe, Germany). Levels of angiopoietin-1 and angiopoietin-2 in sera were measured using ELISA kits (R&D Systems, Minneapolis, MN, USA) according to the manufacturer's instructions.

Normal levels of these cytokines in serum were defined as the mean ± 2 SD of healthy controls.

### Statistical analysis

Results of CXCR1 and CXCR2 expression are presented as medians. Serum levels of IL-8, TNF-α, ANGPT-1 and ANGPT-2 and membrane expression of adhesion molecules are presented as means. Data were analyzed by the Mann-Whitney test and Spearman's rank correlation test using GraphPad Prism 4.03 (GraphPad Software, San Diego, CA, USA). *P *values less than 0.05 were considered significant.

## Results

### Expression of CXCR1 and CXCR2 on the neutrophil membrane is decreased in AAV

**Figure 1:**
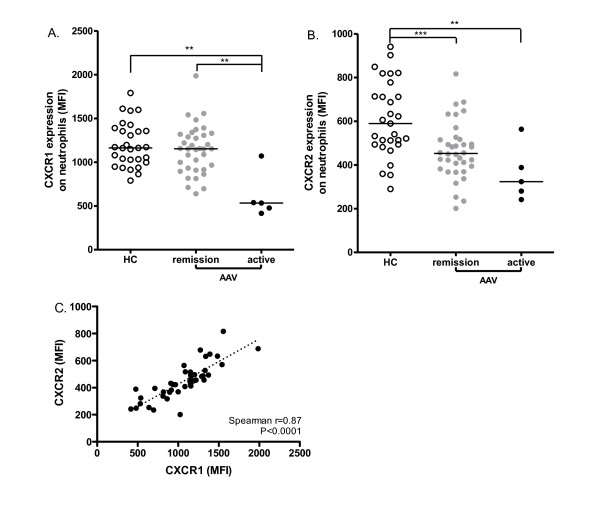
**Down-regulated expression of CXCR1 and CXCR2 on neutrophils in AAV**. Levels of CXCR1 **(A) **and CXCR2 **(B) **expression on the membrane of neutrophils were measured by flow cytometry and compared between AAV patients in remission (*n *= 37), AAV patients with active disease (*n *= 5) and healthy controls (HC, *n *= 30). Expression levels are presented as MFI corrected for nonspecific binding of isotype control antibodies on neutrophils. Horizontal lines represent medians. *, *P *< 0.05; **, *P *< 0.01 ***, *P *< 0.001. Correlation between CXCR1 and CXCR2 expression **(C) **on neutrophils was analyzed in patients in remission and with active disease (*n *= 42, Spearman r = 0.87, *P *< 0.0001). Dotted line represents the best-fit correlation between CXCR1 and CXCR2 expression. AAV, ANCA-associated vasculitides; ANCA, anti-neutrophil cytoplasmic autoantibody; MFI, mean fluorescence intensity.

We first evaluated expression levels of CXCR1 and CXCR2 on neutrophils in AAV patients (remission, *n *= 37; active, *n *= 5) in comparison with healthy blood donors (*n *= 30). Both receptors were highly expressed on the membrane of neutrophils. There was no difference in CXCR1 and CXCR2 expression in patients with different AAV diseases or between patients with MPO- or PR3- ANCA specificity (data not shown). Neutrophils from patients in remission (median 1153, range 640 to approximately 1,986) showed comparable expression of CXCR1 as those from HC (1,163, 790 to approximately 1,790, *P *= 0.3), while neutrophil CXCR1 levels were significantly decreased in patients with active disease (533, 416 to approximately 1,071) compared to either HC (*P *= 0.003) or patients in remission (*P *= 0.0023) (Figure 1A). CXCR2 expression was significantly down-regulated in AAV patients in remission (453, 201 to approximately 817) compared to HC (589, 290 to approximately 941, *P *= 0.0004) and further decreased in active patients (323, 242 to approximately 564) as compared to HC (*P *= 0.0086) (Figure 1B). A significant correlation was observed between the expression of CXCR1 and CXCR2 on neutrophils of AAV patients, regardless of disease activity (Figure 1C, spearman r = 0.87, *P *< 0.0001).

### IL-8 and TNF-α down-regulate CXCR1/CXCR2 from the neutrophil membrane

**Figure 2:**
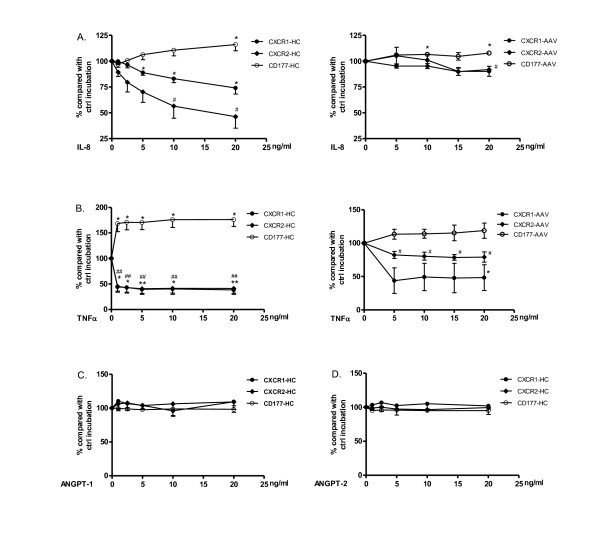
**Down-regulation of CXCR1 and CXCR2 on neutrophils by IL-8 and TNFα**. Isolated neutrophils from HC were pre-incubated with serial doses of IL-8 (**A**, left figure), TNFα (**B**, left figure), ANGPT-1 (**C**) or ANGPT-2 (**D**). Isolated neutrophils from AAV patients were pre-incubated with serial doses of IL-8 (A, right figure) and TNF α (B, right figure). Membrane expression of CXCR1 (black dots) and CXCR2 (black diamonds) was measured by flow cytometry. Expression of CD177 (open dots) was measured in parallel as control for stimulation. Results are presented as percentages compared to control incubation with normal medium (ctrl). CXCR1 and CXCR2 are equally down-regulated by TNFα (overlapping graphs in panel B). Results are the mean (± SD) of three experiments, *, *P *< 0.05; **, *P *< 0.01 (CXCR1 and CD177). #, *P *< 0.01 (CXCR2). AAV, ANCA-associated vasculitides; ANCA, anti-neutrophil cytoplasmic autoantibody; ANGPT, angiopoietin; IL; interleukin; TNF, tumor necrosis factor.

IL-8 and TNF-α are proinflammatory cytokines reported to be elevated in the serum of AAV patients [19-21]. Serum levels of ANGPT-2 have also recently been reported to be increased in AAV and to correlate with disease activity [22]. IL-8 is also produced by endothelial cells stimulated with ANGPT-1 [23]. Therefore, we first tested the influences of these cytokines on CXCR1 and CXCR2 expression on neutrophils of HC. As a result, both IL-8 and TNF-α down-regulated CXCR1 and CXCR2 expression on isolated neutrophils in a dose-dependent manner. IL-8, at a concentration of 5 ng/ml, reduced CXCR2 levels to 61% compared to controls incubated with normal medium, while IL-8 down-regulated membrane expression of CXCR1 to 71% at 20 ng/ml (Figure 2A left figure). TNF-α significantly reduced CXCR1/CXCR2 expression already at a dose of 1 ng/ml (Figure 2B left figure). When tested in parallel, CD177 (NB1) expression was slightly increased by IL-8 and significantly up-regulated by TNF-α, as reported in earlier studies [14,24]. The effects were less obvious when neutrophils of quiescent AAV patients were used. A significant reduction in expression of CXCR1 and CXCR2 was observed only at a concentration of 20 ng/ml of IL-8 (Figure 2A, right figure). Incubation with TNF-α decreased their expression but not to the same extent as seen for HC neutrophils (Figure 2B, right figure). Possibly AAV neutrophils are already pre-primed in the circulation and, therefore, respond less to *in vitro *treatment with cytokines. ANGPT-1 and ANGPT-2 did not affect CXCR1/CXCR2 or CD177 expression on isolated neutrophils (Figure 2C and 2D).

### Serum levels of IL-8, TNF-α, ANGPT-1 and ANGPT-2 in AAV

Levels of the cytokines mentioned above, which may have direct or indirect effects on CXCR1/CXCR2 expression, were evaluated in our patient cohort in comparison with HC.

**Figure 3:**
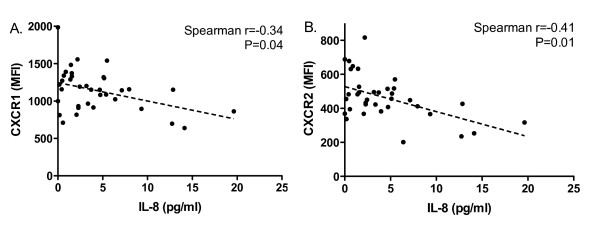
**CXCR1/CXCR2 expression correlates with IL-8 levels in quiescent AAV**. Expression of CXCR1, CXCR2 and levels of IL-8 were measured in parallel in patients in remission (*n *= 37). Membrane expression of CXCR1 and CXCR2 is presented as MFI corrected for nonspecific binding of isotype control antibodies on neutrophils. IL-8 levels are presented as concentration in serum. **A**. Correlation between CXCR1 expression on neutrophils and serum levels of IL-8 (Spearman r = -0.34, *P *= 0.04). **B**. Correlation between CXCR2 expression on neutrophils and serum levels of IL-8 (Spearman r = -0.41, *P *= 0.01). Dotted lines represent the best-fit correlation between expression of CXCR1/2 and IL-8 levels. AAV, ANCA-associated vasculitides; ANCA, anti-neutrophil cytoplasmic autoantibody; IL, interleukin.

**Figure 4:**
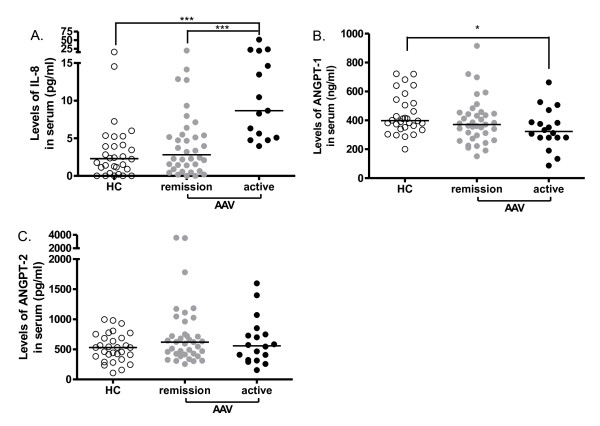
**Levels of IL-8, ANGPT-1 and ANGPT-2 in sera from patients with AAV**. Levels of IL-8 (**A**), ANGPT-1 (**B**) and ANGPT-2 (**C**) in sera of AAV patients in remission (*n *= 37) and active disease (*n *= 18) were measured by ELISA and compared to HC (*n *= 30). Results are presented as the concentration of each cytokine in serum. Bars denote medians, *, *P *< 0.05; ***, *P *< 0.001. AAV, ANCA-associated vasculitides; ANCA, anti-neutrophil cytoplasmic autoantibody; ANGPT, angiopoietin; IL; interleukin.

Defining the normal range as mean ± 2SD of the IL-8 concentration in 30 healthy controls, 5 out of 37 (14%) patients in remission and 10 out of 18 (56%) active patients showed increased IL-8 levels. Levels of IL-8 were not significantly different between patients with quiescent AAV (median 2.81 pg/ml, range 0.0 to approximately 19.64 pg/ml) and healthy controls (2.30 pg/ml, 0 to approximately 15.43 pg/ml). However, a negative correlation was present between CXCR1/CXCR2 expression on neutrophils and levels of IL-8 in patients in remission (Figure 3). A negative correlation was not observed between IL-8 and CXCR1/2 in active AAV, probably due to the small sample size. Nevertheless, in concert with further decreased CXCR1/2 expression, IL-8 levels increased remarkably during active disease (8.67 pg/ml, 3.96 to approximately 51.55 pg/ml), and were significantly higher than levels in HC and patients in remission (Figure 4A).

As for levels of ANGPT-1, no significant difference was observed between HC and patients in remission. The median level of ANGPT-1 in active patients was lower than in HC (Figure 4B). Levels of ANGPT-2 were not increased in patients in remission nor in patients with active disease (Figure 4C). TNF-α was not detectable in sera of either HC or patient groups.

### Neutrophil recruitment after CXCR1/CXCR2 blockade

To further clarify possible consequences of CXCR1 and CXCR2 down-regulation on neutrophils, we investigated neutrophil adhesion and transmigration through a glomerular endothelial monolayer. Neutrophils were pretreated with repertaxin, mimicking decreased efficiency of IL-8 receptors, or stimulated with IL-8/TNF-α, both of which lead to decline of CXCR1 and CXCR2 expression.

**Figure 5:**
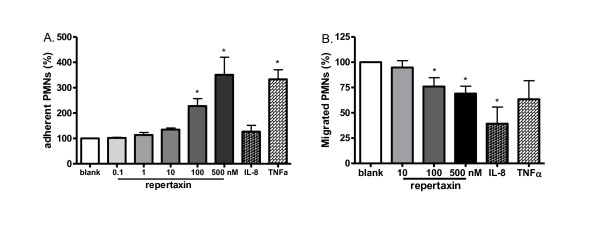
**CXCR1/CXCR2 blockade enhances neutrophil adhesion and inhibits neutrophil transmigration through an endothelial cell monolayer**. Isolated neutrophils from HC were pre-incubated with serial doses of repertaxin, IL-8 (10 ng/ml) or TNFα (2 ng/ml) before being loaded onto a glomerular endothelial monolayer or a transwell insert. After co-incubation of pretreated neutrophils with glomerular endothelial cells for 30 minutes for the adhesion assay (**A**, *n *= 5) or with transwell inserts coated with endothelial cells for 2 hours (**B**, *n *= 6), the number of adherent or migrated neutrophils were quantified by MPO activity assay. Results are presented as the percentages of adherent or migrated neutrophils after stimulation compared to control incubation. *, *P *< 0.05. HC, healthy control; IL; interleukin; MPO, myeloperoxidase; TNF, tumor necrosis factor.

Glomerular endothelial cell layers were stimulated with 10 ng/ml of TNF-α for 4 hours prior to the adhesion or migration assay to mimic activated glomerular endothelial cells as occur in AAV. Preincubation with repertaxin inhibited neutrophil migration in a dose-dependent way. Migration was reduced to 76% by 100 nM of repertaxin, significantly lower than control incubated neutrophils. IL-8 preincubation reduced the number of migrated neutrophils to 39%. Although incubation with TNF-α resulted in a significantly reduced expression of CXCR1/CXCR2 on the membrane of neutrophils, migration was not significantly influenced, and numbers of migrated neutrophils were comparable with control incubation (Figure 5B).

Neutrophils preincubated with 2 ng/ml TNF-α showed enhanced adhesion to glomerular endothelium compared to control incubation. IL-8 (up to 20 ng/ml), however, did not show a remarkable effect on neutrophil adhesion. Blocking CXCR1 and CXCR2 with repertaxin also increased the number of adherent neutrophils dose-dependently, from 135% with 10 nM of repertaxin up to more than 300% at 0.5 μM as compared to cells without stimulation (Figure 5A).

## Discussion

This is the first study, to our knowledge, reporting decreased expression of CXCR1 and CXCR2 on circulating neutrophils in AAV, potentially induced by proinflammatory cytokines such as IL-8. Functional deficiency of CXCR1/CXCR2, induced by specific inhibition of these receptors with repertaxin, increased neutrophil adhesion and impaired migration through a glomerular endothelium monolayer. Down-regulation of CXCR1 and CXCR2 may retain activated neutrophils within the vessel wall, allowing interaction with circulating ANCA, so leading to vascular damage *in situ*. These findings highlight a chemokine-receptor-based mechanism of inflammation confined within the vessel wall and would further explain the role of neutrophils in the pathogenesis of AAV.

CXCR1 and CXCR2 are not stably expressed on the neutrophil membrane. During chemotaxis, ligand-mediated internalization and recycling of these chemokine receptors is proposed as a self-limiting mechanism allowing neutrophils, to retain *in situ *by desensitization to their ligands[25]. However, this physiological process is probably abused in AAV, and down-regulated CXCR1/CXCR2 expression may desensitize circulating neutrophils to chemotactic signals before they migrate into the tissue resulting in accumulation of neutrophils within the vessel wall.

Our *in vitro *experiments confirmed this hypothesis by showing significantly increased adhesion and decreased migration of neutrophils through glomerular endothelium after CXCR1 and CXCR2 blockade. This is in line with our previous observation that, in a model of MPO-ANCA-associated vasculitis, repertaxin treated mice were not protected from hematuria, albuminuria or glomerular crescent formation. Although some neutrophil migration through the endothelial monolayer in our *in vitro *experiments can not completely be ruled out, we observed a larger number of neutrophils accumulating in glomeruli in comparison to untreated mice [26]. Besides, CXCR1/CXCR2-knockout mice showed a deficiency in neutrophil migration and bacterial clearance in a urinary tract infection model, and decline in CXCR1/CXCR2 expression may worsen outcome of infections due to impaired neutrophil recruitment [27-31].

Notably, down-regulation of CXCR1 and CXCR2 from the neutrophil surface may not be specific for AAV. As IL-8 is a potential inducer of decreased CXCR1/2 expression, increased levels of circulating IL-8 in other clinical conditions may also result in a similar decline of CXCR1/2 expression. In addition, our results may reflect a state of neutrophil activation mediated by IL-8 or other proinflammatory cytokines, such as TNFα, as has been described in AAV by Muller Kobold *et al. *previously [32]. In the presence of ANCA, however, pre-activated neutrophils lacking CXCR1/2 expression can be confined in the microvasculature and fully activated by ANCA.

The role of CXCR1 and CXCR2 in neutrophil adhesion has not been clearly delineated. Blocking of IL-8-receptors showed an inhibitory effect on IL-8-induced neutrophil adhesion in an early study [33]. The underlying mechanisms are not fully elucidated so far. A recent study conducted by Cohen-Hillel *et al. *showed that migratory desensitization triggers over-activation of focal adhesion kinase (FAK), which leads to increased neutrophil adhesion [34]. The latter mechanism is likely operative in AAV, where neutrophils are migratory desensitized under continuous exposure to low-dose IL-8 [20]. Besides, in our experiments, neutrophils pretreated with repertaxin did show significantly increased adhesion on an activated CiGEnC monolayer. As endothelial cells might also be actively involved in neutrophil-endothelial interaction during inflammation, this adhesive effect may also depend on endothelial phenotypes and microenvironment, explaining why Coelho *et al. *observed short-term suppression of neutrophil adhesion to synovial endothelium after repertaxin treatment in a mouse model of arthritis [35]. Glomerular endothelial cells (ECs) have unique features and differ from synovial ECs in many ways, such as expression profile of adhesion molecules [36]. Neutrophil recruitment in an *in vitro *model of rheumatoid arthritis only occurs in co-cultures of ECs with synovial fibroblasts but not with control tissues [37], suggesting that immune regulators in the microenvironment also play an important role in this process. We are aware of the fact that the cells used in our study were glomerular endothelial cells that were immortalized, and therefore seem artificial. However these cells were shown to have retained morphological features of early-passage primary culture glomerular ECs and they expressed EC adhesion molecules and other specific features [13].

Since CXCR1 and CXCR2 are internalized upon ligation and IL-8 has been shown to be significantly elevated in AAV patients [20], we speculate that IL-8 is one of the factors inducing decreased expression of these receptors. Indeed, we found that CXCR1 and CXCR2 expression was remarkably lower in patients with active AAV than in HC. Decreased expression was accompanied by significantly elevated serum levels of IL-8 in active AAV. Moreover, a negative correlation was observed between CXCR1/CXCR2 expression and serum IL-8 levels in patients with quiescent disease. As an indirect evidence of this relationship, a negative effect of serum IL-8 on neutrophil recruitment has been demonstrated in earlier studies which was supposed to be caused by down-regulation of IL-8 receptors [38,39]. Cockwell *et al. *proposed a similar scenario in AAV by showing that ANCA-activated neutrophils produce IL-8 and that neutrophils accumulate locally in the glomerular capillary loops but poorly penetrate into IL-8 enriched tissues as observed in renal biopsies of AAV patients [40]. From the data of the present study, a relationship is suggested between circulating IL-8 levels, decrease in CXCR1/CXCR2 expression and neutrophil accumulation in the microvasculature in AAV.

Circulating IL-8 can be produced by ANCA-activated neutrophils and endothelial cells stimulated by IL-1, TNF-α or PR3-ANCA [40-42]. Although circulating IL-8 levels were not significantly elevated in patients during remission, it should be noted that a low-level of immune activation is present during quiescent disease as well, including minor increases in IL-8 levels [43].

CXCR1 and CXCR2 expression can also be regulated, via enzymatic cleavage via metalloproteinases [44], by other immunomodulators such as TNF-α which have been detected at inflammatory sites in AAV and prime neutrophils for ANCA-induced activation [20,45-47]. Technical limitations to measure TNFα in serum could have hampered the revelation of the relationship between TNFα levels and CXCR1/2 expression. As shown in the current study, TNF-α, up to a concentration of 20 ng/ml, was more efficient than IL-8 in down-regulating CXCR1/CXCR2 *in vitro*. Therefore, although TNF-α could not be detected in sera, there is still the possibility that increased levels of TNF-α, especially during active disease, lead to a decrease in CXCR1 and CXCR2 expression.

It needs to be mentioned that the number of patients with active AAV included in the current study was relatively small, and so does not allow firm conclusions as to the correlation between neutrophil CXCR1 and CXCR2 expression and disease activity. Such a correlation is, however, suggested by the further decrease of CXCR1 and CXCR2 expression that we observed during active disease. Furthermore, immunohistochemical studies are needed to show lack of CXCR1 and CXCR2 expression on neutrophils recruited to the vascular wall in AAV. Such data could further strengthen the role of CXCR1 and CXCR2 down-regulation in AAV.

## Conclusions

Expression of CXCR1 and CXCR2 on neutrophils is down-regulated in AAV patients, potentially induced by circulating IL-8. Decreased expression of these chemokine receptors may enhance neutrophil adhesion and impede neutrophil migration through activated glomerular endothelium, which may account for neutrophil accumulation and persistent ANCA-induced inflammation in the vessel wall.

## Abbreviations

AAV: ANCA-associated vasculitides; ABTS: 2.2'-azino-*bis*-3-ethylbenzothiazoline-6-sulfonic acid; ANCA: anti-neutrophil cytoplasmic autoantibody; ANGPT: angiopoietin; APC: allophycocyanin; BVAS: Birmingham Vasculitis Activity Score; CiGEnC: conditionally immortalized glomerular endothelial cell; CSS: Churg Strauss syndrome; CYC: cyclophosphamide; EC: endothelial cell; FCS: fetal calf serum; FITC: fluorescein isothiocyanate; GPA: granulomatosis with polyangiitis; HBSS: Hanks' balanced salt solution; HC: healthy control; IL: interleukin; MFI: mean fluorescence intensity; MMF: mecophenolate mophetil; MPA: microscopic polyangiitis; MPO: myeloperoxidase; PE: phycoerythrin; PR3: proteinase 3; TEER: trans-endothelial electrical resistance; TMB: tetramethyl-benzidin; TNF: tumor necrosis factor; VEGF: vascular endothelial growth factor.

## Competing interests

The authors declare that they have no competing interests.

## Authors' contributions

NH made substantial contributions to conception and design of this study, acquisition, analysis and interpretation of data and drafted the manuscript; JW and PH were involved in the study design, data interpretation and revised the manuscript critically; WHA, MGH and BD contributed in the data acquisition; AR and CAS collected patient materials from out-patient clinic; PWM and SCS kindly provided the human glomerular endothelial cell line for this research; CGMK participated in the study design and data analysis and interpretation and revised the manuscript carefully and critically. All authors read and approved the final manuscript.

## References

[B1] Jennette JC Falk RJ Small-vessel vasculitis N Engl J Med 1997 337 1512 1523 10.1056/NEJM199711203372106 9366584

[B2] Kallenberg CG Heeringa P Stegeman CA Mechanisms of disease: pathogenesis and treatment of ANCA-associated vasculitides Nat Clin Pract Rheumatol 2006 2 661 670 10.1038/ncprheum0355 17133251

[B3] Falk RJ Terrell RS Charles LA Jennette JC Anti-neutrophil cytoplasmic autoantibodies induce neutrophils to degranulate and produce oxygen radicals in vitro Proc Natl Acad Sci USA 1990 87 4115 4119 10.1073/pnas.87.11.4115 2161532 PMC54058

[B4] Savage CO Pottinger BE Gaskin G Pusey CD Pearson JD Autoantibodies developing to myeloperoxidase and proteinase 3 in systemic vasculitis stimulate neutrophil cytotoxicity toward cultured endothelial cells Am J Pathol 1992 141 335 342 1323218 PMC1886603

[B5] Xiao H Heeringa P Liu Z Huugen D Hu P Maeda N Falk RJ Jennette JC The role of neutrophils in the induction of glomerulonephritis by anti-myeloperoxidase antibodies Am J Pathol 2005 167 39 45 10.1016/S0002-9440(10)62951-3 15972950 PMC1603451

[B6] Murphy PM Neutrophil receptors for interleukin-8 and related CXC chemokines Semin Hematol 1997 34 311 318 9347581

[B7] Pham CT Neutrophil serine proteases: specific regulators of inflammation Nat Rev Immunol 2006 6 541 550 10.1038/nri1841 16799473

[B8] Abdel-Salam B Iking-Konert C Schneider M Andrassy K Hansch GM Autoantibodies to neutrophil cytoplasmic antigens (ANCA) do not bind to polymorphonuclear neutrophils in blood Kidney Int 2004 66 1009 1017 10.1111/j.1523-1755.2004.00849.x 15327394

[B9] Brachemi S Mambole A Fakhouri F Mouthon L Guillevin L Lesavre P Halbwachs-Mecarelli L Increased membrane expression of proteinase 3 during neutrophil adhesion in the presence of anti proteinase 3 antibodies J Am Soc Nephrol 2007 18 2330 2339 10.1681/ASN.2006121309 17634439

[B10] Reumaux D Vossebeld PJ Roos D Verhoeven AJ Effect of tumor necrosis factor-induced integrin activation on Fc gamma receptor II-mediated signal transduction: relevance for activation of neutrophils by anti-proteinase 3 or anti-myeloperoxidase antibodies Blood 1995 86 3189 3195 7579414

[B11] Van Rossum AP Van Der Geld YM Limburg PC Kallenberg CG Human anti-neutrophil cytoplasm autoantibodies to proteinase 3 (PR3-ANCA) bind to neutrophils Kidney Int 2005 68 537 541 10.1111/j.1523-1755.2005.00431.x 16014030

[B12] Jennette JC Falk RJ Andrassy K Bacon PA Churg J Gross WL Hagen EC Hoffman GS Hunder GG Kallenberg CG Nomenclature of systemic vasculitides. Proposal of an international consensus conference Arthritis Rheum 1994 37 187 192 10.1002/art.1780370206 8129773

[B13] Satchell SC Tasman CH Singh A Ni L Geelen J von Ruhland CJ O'Hare MJ Saleem MA van den Heuvel LP Mathieson PW Conditionally immortalized human glomerular endothelial cells expressing fenestrations in response to VEGF Kidney Int 2006 69 1633 1640 10.1038/sj.ki.5000277 16557232

[B14] Hu N Westra J Huitema MG Bijl M Brouwer E Stegeman CA Heeringa P Limburg PC Kallenberg CG Coexpression of CD177 and membrane proteinase 3 on neutrophils in antineutrophil cytoplasmic autoantibody-associated systemic vasculitis: anti-proteinase 3-mediated neutrophil activation is independent of the role of CD177-expressing neutrophils Arthritis Rheum 2009 60 1548 1557 10.1002/art.24442 19404956

[B15] Louis NA Campbell E Colgan SP Quinn MT, Deleo FR, Bokoch GM Model systems to investigate neutrophil adhesion and chemotaxis Neutrophil methods and protocols 2007 Totowa: Humana Press 257 272

[B16] Zouki C Beauchamp M Baron C Filep JG Prevention of in vitro neutrophil adhesion to endothelial cells through shedding of L-selectin by C-reactive protein and peptides derived from C-reactive protein J Clin Invest 1997 100 522 529 10.1172/JCI119561 9239398 PMC508218

[B17] Bertini R Allegretti M Bizzarri C Moriconi A Locati M Zampella G Cervellera MN Di Cioccio V Cesta MC Galliera E Martinez FO Di Bitondo R Troiani G Sabbatini V D'Anniballe G Anacardio R Cutrin JC Cavalieri B Mainiero F Strippoli R Villa P Di Girolamo M Martin F Gentile M Santoni A Corda D Poli G Mantovani A Ghezzi P Colotta F Noncompetitive allosteric inhibitors of the inflammatory chemokine receptors CXCR1 and CXCR2: prevention of reperfusion injury Proc Natl Acad Sci USA 2004 101 11791 11796 10.1073/pnas.0402090101 15282370 PMC511013

[B18] Westra J Brouwer E van Roosmalen IA Doornbos-van der Meer B van Leeuwen MA Posthumus MD Kallenberg CG Expression and regulation of HIF-1alpha in macrophages under inflammatory conditions; significant reduction of VEGF by CaMKII inhibitor BMC Musculoskelet Disord 2010 11 61 10.1186/1471-2474-11-61 20353560 PMC2851671

[B19] Jonasdottir O Petersen J Bendtzen K Tumour necrosis factor-alpha (TNF), lymphotoxin and TNF receptor levels in serum from patients with Wegener's granulomatosis APMIS 2001 109 781 786 10.1034/j.1600-0463.2001.d01-146.x 11900058

[B20] Ohlsson S Wieslander J Segelmark M Circulating cytokine profile in anti-neutrophilic cytoplasmatic autoantibody-associated vasculitis: prediction of outcome? Mediators Inflamm 2004 13 275 283 10.1080/09629350400003100 15545059 PMC1781567

[B21] Tesar V Masek Z Rychlik I Merta M Bartunkova J Stejskalova A Zabka J Janatkova I Fucikova T Dostal C Becvar R Cytokines and adhesion molecules in renal vasculitis and lupus nephritis Nephrol Dial Transplant 1998 13 1662 1667 10.1093/ndt/13.7.1662 9681708

[B22] Kumpers P Hellpap J David S Horn R Leitolf H Haller H Haubitz M Circulating angiopoietin-2 is a marker and potential mediator of endothelial cell detachment in ANCA-associated vasculitis with renal involvement Nephrol Dial Transplant 2009 24 1845 1850 10.1093/ndt/gfn755 19164323

[B23] Lemieux C Maliba R Favier J Theoret JF Merhi Y Sirois MG Angiopoietins can directly activate endothelial cells and neutrophils to promote proinflammatory responses Blood 2005 105 1523 1530 10.1182/blood-2004-09-3531 15498854

[B24] Bauer S Abdgawad M Gunnarsson L Segelmark M Tapper H Hellmark T Proteinase 3 and CD177 are expressed on the plasma membrane of the same subset of neutrophils J Leukoc Biol 2007 81 458 464 10.1189/jlb.0806514 17077162

[B25] Rose JJ Foley JF Murphy PM Venkatesan S On the mechanism and significance of ligand-induced internalization of human neutrophil chemokine receptors CXCR1 and CXCR2 J Biol Chem 2004 279 24372 24386 10.1074/jbc.M401364200 15028716

[B26] van der Veen BS Petersen AH Belperio JA Satchell SC Mathieson PW Molema G Heeringa P Spatiotemporal expression of chemokines and chemokine receptors in experimental anti-myeloperoxidase antibody-mediated glomerulonephritis Clin Exp Immunol 2009 158 143 153 10.1111/j.1365-2249.2009.03993.x 19737241 PMC2759069

[B27] Godaly G Hang L Frendeus B Svanborg C Transepithelial neutrophil migration is CXCR1 dependent in vitro and is defective in IL-8 receptor knockout mice J Immunol 2000 165 5287 5294 11046063 10.4049/jimmunol.165.9.5287

[B28] Mintz R Garty BZ Meshel T Marcus N Katanov C Cohen-Hillel E Ben Baruch A Reduced expression of chemoattractant receptors by polymorphonuclear leukocytes in Hyper IgE Syndrome patients Immunol Lett 2010 130 97 106 10.1016/j.imlet.2009.12.006 20005258

[B29] Olszyna DP Florquin S Sewnath M Branger J Speelman P van Deventer SJ Strieter RM van der Poll T CXC chemokine receptor 2 contributes to host defense in murine urinary tract infection J Infect Dis 2001 184 301 307 10.1086/322030 11443555

[B30] Slocombe RF Malark J Ingersoll R Derksen FJ Robinson NE Importance of neutrophils in the pathogenesis of acute pneumonic pasteurellosis in calves Am J Vet Res 1985 46 2253 2258 4073635

[B31] Svensson M Irjala H Svanborg C Godaly G Effects of epithelial and neutrophil CXCR2 on innate immunity and resistance to kidney infection Kidney Int 2008 74 81 90 10.1038/ki.2008.105 18401338

[B32] Muller Kobold AC Mesander G Stegeman CA Kallenberg CG Tervaert JW Are circulating neutrophils intravascularly activated in patients with anti-neutrophil cytoplasmic antibody (ANCA)-associated vasculitides? Clin Exp Immunol 1998 114 491 499 10.1046/j.1365-2249.1998.00748.x 9844062 PMC1905131

[B33] Casilli F Bianchini A Gloaguen I Biordi L Alesse E Festuccia C Cavalieri B Strippoli R Cervellera MN Di Bitondo R Ferretti E Mainiero F Bizzarri C Colotta F Bertini R Inhibition of interleukin-8 (CXCL8/IL-8) responses by repertaxin, a new inhibitor of the chemokine receptors CXCR1 and CXCR2 Biochem Pharmacol 2005 69 385 394 10.1016/j.bcp.2004.10.007 15652230

[B34] Cohen-Hillel E Yron I Meshel T Ben Baruch A Interleukin 8 and cell migration to inflammatory sites: the regulation of focal adhesion kinase under conditions of migratory desensitization Isr Med Assoc J 2007 9 579 583 17877062

[B35] Coelho FM Pinho V Amaral FA Sachs D Costa VV Rodrigues DH Vieira AT Silva TA Souza DG Bertini R Teixeira AL Teixeira MM The chemokine receptors CXCR1/CXCR2 modulate antigen-induced arthritis by regulating adhesion of neutrophils to the synovial microvasculature Arthritis Rheum 2008 58 2329 2337 10.1002/art.23622 18668539

[B36] Savage CO Brooks CJ Adu D Richards G Howie AJ Cell adhesion molecule expression within human glomerular and kidney organ culture J Pathol 1997 181 111 115 10.1002/(SICI)1096-9896(199701)181:1<111::AID-PATH698>3.0.CO;2-9 9072012

[B37] Lally F Smith E Filer A Stone MA Shaw JS Nash GB Buckley CD Rainger GE A novel mechanism of neutrophil recruitment in a coculture model of the rheumatoid synovium Arthritis Rheum 2005 52 3460 3469 10.1002/art.21394 16255036 PMC3119436

[B38] Hechtman DH Cybulsky MI Fuchs HJ Baker JB Gimbrone MA Jr Intravascular IL-8. Inhibitor of polymorphonuclear leukocyte accumulation at sites of acute inflammation J Immunol 1991 147 883 892 1650387

[B39] Simonet WS Hughes TM Nguyen HQ Trebasky LD Danilenko DM Medlock ES Long-term impaired neutrophil migration in mice overexpressing human interleukin-8 J Clin Invest 1994 94 1310 1319 10.1172/JCI117450 7521886 PMC295217

[B40] Cockwell P Brooks CJ Adu D Savage CO Interleukin-8: A pathogenetic role in antineutrophil cytoplasmic autoantibody-associated glomerulonephritis Kidney Int 1999 55 852 863 10.1046/j.1523-1755.1999.055003852.x 10027922

[B41] Mayet W Schwarting A Barreiros AP Schlaak J Neurath M Anti-PR-3 antibodies induce endothelial IL-8 release Eur J Clin Invest 1999 29 973 979 10.1046/j.1365-2362.1999.00555.x 10583443

[B42] Strieter RM Kunkel SL Showell HJ Remick DG Phan SH Ward PA Marks RM Endothelial cell gene expression of a neutrophil chemotactic factor by TNF-alpha, LPS, and IL-1 beta Science 1989 243 1467 1469 10.1126/science.2648570 2648570

[B43] Abdulahad WH Stegeman CA Limburg PC Kallenberg CG Skewed distribution of Th17 lymphocytes in patients with Wegener's granulomatosis in remission Arthritis Rheum 2008 58 2196 2205 10.1002/art.23557 18576340

[B44] Khandaker MH Mitchell G Xu L Andrews JD Singh R Leung H Madrenas J Ferguson SS Feldman RD Kelvin DJ Metalloproteinases are involved in lipopolysaccharide- and tumor necrosis factor-alpha-mediated regulation of CXCR1 and CXCR2 chemokine receptor expression Blood 1999 93 2173 2185 10090924

[B45] Chan AT Flossmann O Mukhtyar C Jayne DR Luqmani RA The role of biologic therapies in the management of systemic vasculitis Autoimmun Rev 2006 5 273 278 10.1016/j.autrev.2006.01.003 16697969

[B46] Huugen D Xiao H van Esch A Falk RJ Peutz-Kootstra CJ Buurman WA Tervaert JW Jennette JC Heeringa P Aggravation of anti-myeloperoxidase antibody-induced glomerulonephritis by bacterial lipopolysaccharide: role of tumor necrosis factor-alpha Am J Pathol 2005 167 47 58 10.1016/S0002-9440(10)62952-5 15972951 PMC1603449

[B47] Noronha IL Kruger C Andrassy K Ritz E Waldherr R In situ production of TNF-alpha, IL-1 beta and IL-2R in ANCA-positive glomerulonephritis Kidney Int 1993 43 682 692 10.1038/ki.1993.98 8455368

